# Exploring Phytochemicals for Combating Antibiotic Resistance in Microbial Pathogens

**DOI:** 10.3389/fphar.2021.720726

**Published:** 2021-07-21

**Authors:** Tushar Khare, Uttpal Anand, Abhijit Dey, Yehuda G. Assaraf, Zhe-Sheng Chen, Zhijun Liu, Vinay Kumar

**Affiliations:** ^1^ Department of Biotechnology, Modern College of Arts, Science and Commerce (Savitribai Phule Pune University), Pune, India; ^2^ Department of Environmental Science, Savitribai Phule Pune University, Pune, India; ^3^ Department of Life Sciences and the National Institute for Biotechnology in the Negev, Ben-Gurion University of the Negev, Beer-Sheva, Israel; ^4^ Ethnopharmacology and Natural Product Research Laboratory, Department of Life Sciences, Presidency University, Kolkata, India; ^5^ The Fred Wyszkowski Cancer Research Laboratory, Department of Biology, Technion-Israel Institute of Technology, Haifa, Israel; ^6^ Department of Pharmaceutical Sciences, College of Pharmacy and Health Sciences, St. John’s University, Queens, NY, United States; ^7^ Department of Microbiology, Weifang Medical University, Weifang, China

**Keywords:** antibiotics, antimicrobial, efflux pumps, medicinal plants, multidrug resistance, phytomolecules, drug resistance reversal agents

## Abstract

Antibiotic resistance or microbial drug resistance is emerging as a serious threat to human healthcare globally, and the multidrug-resistant (MDR) strains are imposing major hurdles to the progression of drug discovery programs. Newer antibiotic-resistance mechanisms in microbes contribute to the inefficacy of the existing drugs along with the prolonged illness and escalating expenditures. The injudicious usage of the conventional and commonly available antibiotics in human health, hygiene, veterinary and agricultural practices is proving to be a major driver for evolution, persistence and spread of antibiotic-resistance at a frightening rate. The drying pipeline of new and potent antibiotics is adding to the severity. Therefore, novel and effective new drugs and innovative therapies to treat MDR infections are urgently needed. Apart from the different natural and synthetic drugs being tested, plant secondary metabolites or phytochemicals are proving efficient in combating the drug-resistant strains. Various phytochemicals from classes including alkaloids, phenols, coumarins, terpenes have been successfully demonstrated their inhibitory potential against the drug-resistant pathogens. Several phytochemicals have proved effective against the molecular determinants responsible for attaining the drug resistance in pathogens like membrane proteins, biofilms, efflux pumps and bacterial cell communications. However, translational success rate needs to be improved, but the trends are encouraging. This review highlights current knowledge and developments associated challenges and future prospects for the successful application of phytochemicals in combating antibiotic resistance and the resistant microbial pathogens.

## Introduction

The injudicious use of antibiotics for human healthcare has led to the fast emergence, persistence and spread of the antibiotic or antimicrobial resistance, a condition in which microbes develop resistance against the spectrum of conventional antibiotics, also known as multidrug resistance and these microbes are typically known as antibiotic- or drug-resistant pathogens (Yu et al., 2020). It has left the medical community with a shrunken pool of therapeutic options against these strains. The infections associated with antibiotic resistant pathogens are usually accompanied by substantial morbidity and mortality along with the massive economic burden on global healthcare (Centers for Disease Control and Prevenation, 2019). Unfortunately, MDR pathogens are receiving progressive identification in community-acquired infections (Koulenti et al., 2020). Along with the excessive antibiotics usage in human healthcare, antibiotics used in veterinary also owe to the emergence of antibiotic resistance (Smet et al., 2009). Further, subsequent contamination of soil, sediments and water bodies accelerate the process of development and spread of antibiotic resistant strains in the environment (Gothwal and Shashidhar, 2015; Ben Said et al., 2016). Besides, the waste generated by pharma-industries, hospitals as well as livestock producers carries the unmonitored quantities of antimicrobials, which stimulate the multi-drug resistance in the environment, with prospective resistome-spill over to humans and animals (Oyekale and Oyekale, 2017; World Bank, 2017). Looking at the speed of drug resistance emergence and its serious threat to the global health, development of new drugs to treat such infections is the top priority for the World Health Organization (WHO) (The Lancet Infectious Diseases, 2017).

The WHO has published the first-ever list of antibiotic-resistant *priority pathogens*, describing a catalogue of twelve bacterial families posing the greatest threat to human health (World Health Organization, 2017). The list covers the resistant pathogens in three tiers as per the urgency of new antibiotics. The first group represents critical priority pathogens, which includes *Acinetobacter baumannii*, *Pseudomonas aeruginosa* and members of *Enterobacteriaceae* (*Klebsiella*, *Escherichia coli*, *Serratia* and *Proteus*). The second group is a high priority group with the pathogens including *Enterococcus faecium*, *Staphylococcus aureus*, *Helicobacter pylori*, *Campylobacter* spp., *Salmonellae*, and *Neisseria gonorrhoeae*. The last group contains medium priority pathogens such as *Streptococcus pneumoniae*, *Haemophilus influenzae*, and *Shigella* spp. (World Health Organization, 2017). Based on their drug-resistance profile, pathogens can be categorized as multidrug resistant (MDR), extensively drug-resistant (XDR) or pandrug-resistant (PDR) as defined by European Centre for Disease Prevention and Control (ECDC) and the Centers for Disease Control and Prevention (CDC) (Magiorakos et al., 2012). These MDR pathogens are typically associated with nosocomial infections and their trends have been observed concerning certain medical conditions. For instance, retrospective analysis based on five-year single cohort epidemiological observations of MDR infections in oncological patients showed that the carbapenem-resistant *Klebsiella pneumoniae*, methicillin-resistant *Staphylococcus aureus* (MRSA) and carbapenem-resistant *Acinetobacter baumannii* were most frequently isolated strains, with 32% infection-associated mortality (Perdikouri et al., 2019). Diabetic foot infections have exhibited an association with biofilm-forming resistant strains (Vatan et al., 2018). Therefore, conducting a simultaneous process of curing the disease as well as combating the related MDR pathogens is very complex and challenging.

There is an urgent need for new antibacterial agents to fight against the threat of microbial antibiotic resistance. Unfortunately, the progress in this direction is notably slow (Fischbach and Walsh, 2009). Out of the alternative and effective options against MDR pathogens, plants might hold the key in providing the enormous range of the chemotherapeutics in form of their secondary metabolites with an ability to tackle bacterial infections (Anand et al., 2019; Anand et al., 2020). These secondary metabolites or phytochemicals include the members of alkaloids, flavonoids, quinones, coumerins and many more (Mbaveng et al., 2015; Anand et al., 2019; Anand et al., 2020; Mohammed et al., 2021). The current review depicts the potential role of the plant secondary metabolites or phyto-antimicrobials against the antibiotic resistant pathogens. The review incorporates the major molecular determinants of the antibiotic resistance and the bactericidal as well as antibiotic-resistance reversal potentials of phytochemicals. The mini review describes the successful attempts of the applications of phytochemicals in the fight against the antibiotic resistant bacterial pathogens, especially of clinical origin.

## Major Determinants of Antibiotic Resistance

**FIGURE 1:**
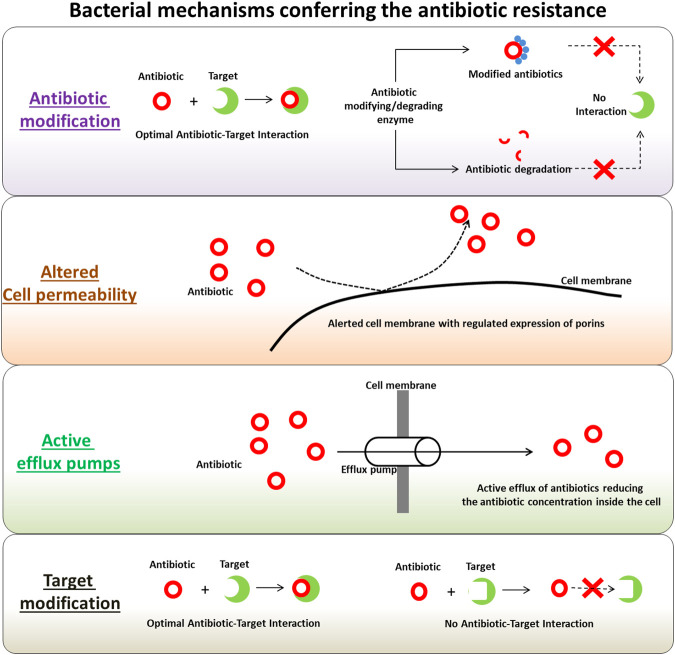
Summary of bacterial mechanisms conferring the antibiotic resistance.

Several mechanisms have been identified in the microbial pathogens involved in their self-defense machinery active against the antimicrobial drugs, antibiotics and pesticides. These mechanisms are often active simultaneously in pathogenic microbes, especially with antibiotic resistant phenotypes, ensuring their protection against a wide range of antibiotics. With each application of treatment options, the likelihood of being less effective against both intended and other targets increases when used in the future. The applications of antimicrobial drugs trigger a complex interaction involving these drug-resistance determinants. Interestingly, these mechanisms have also been observed in antibiotic-producing organisms and the genetic determinants coding for the defense machinery are found in clusters with antibiotic biosynthesis-related genes. This forms a complex co-regulated network in pathogens (Mak et al., 2014). These regulated machineries are which mediates the pathogen defense mechanistically diverse and possibly one of the main causes of the origin of antibiotic resistance (Munita and Arias, 2016). Figure 1 depicts the major tactics of antibiotic-resistant strains against wide-spectrum and class of antibiotics, as discussed in the following sections. The evolution of antibiotic resistance can be considered into two categories, the first one being the molecular redundancy, where organisms try to adapt their physiology to newer (and challenging) environments on receiving the signals, and second being the molecular infidelity, where stressed microbial cells become more receptive to foreign DNA besides certain stresses can also induce rapid mutations. Microbes are highly adaptable living organisms with tremendous abilities to mutate in response to their environments. These mutated bacterial strains produce populations with drug-resistance characteristics and can transfer drug-resistance carrying genes and genetic elements to other non-resistant bacteria in the surroundings, through horizontal gene transfer mechanisms. Following sections discuss the main antibiotic resistance determinants in bacteria with antibiotic resistance phenotypes.

### Modification or Destruction of Antibiotics

One of the effective strategies in bacteria for coping up with the antibiotics is production of enzymes which modifies the active moieties of the antibiotics via addition of chemical groups or which destroys the antibiotics. This prevents the interaction between the antibiotics and their respective targets in bacteria (Fischbach and Walsh, 2009). The enzymes with capability to induce chemical alterations in antibiotics are well-known in both Gram-negative and Gram-positive pathogens. Most of these antibiotics impacted by the chemical alterations exert their mode of action via inhibiting the protein synthesis at ribosomal level (Wilson, 2014). Most frequently observed chemical modifications of the antibiotics include phosphorylation (chloramphenicol, aminoglycosides), adenylation (lincosamides, aminoglycosides) and acetylation (streptogramins, chloramphenicol, aminoglycosides) of the antibiotics. Some of the most studied examples of antibiotic modifying enzyme include aminoglycoside-modifying enzymes (AMEs) and chloramphenicol acetyltransferases, which are usually harbored in the mobile genetic elements. Irrespective of the biochemical reactions, this alteration results in steric hindrance, which in turn reduces the antibiotic avidity for its target, and thus increasing the minimal inhibitory concentrations (MIC) during the treatment (Munita and Arias, 2016). On the other hand, there is a production of potent bacterial enzymes, good enough to destruct the applied antibiotics. These enzymes typically belong to the class of β-lactamases and destroy the amide bond of the β-lactam ring, making the antimicrobial ineffective. Multiple form of these enzymes usually denoted as ‘bla’, are disseminated on the mobile genetic elements (Tooke et al., 2019). The β-lactamases can be classified in two different ways; either based on their amino acid sequences or based on their biochemical function and substrate specificities (Bush and Jacoby, 2010; Bush, 2013). The β-lactamases are progressively becoming the prime choice for the empirical therapy of nosocomial infections and their interactions with lactams like carbapenems is of a particular interest while studying the resistance (Tooke et al., 2019). Besides this, there are some other types of enzymes, categorized as Extended Spectrum β-lactamases (ESBLs) and having an ability to hydrolyze and deactivate the antibiotics such as penicillins, third-generation cephalosporins and monobactams. The spread of Extended Spectrum β-lactamases is strongly associated with the conjugative plasmids (ur Rahman, 2018).

### Cell Permeability and Drug-Efflux Pumps

In order to exert the effective antibiotic activity, it is necessary for the antibiotics to enter the bacterial cell via penetrating the cell membrane and further interact with the respective cellular targets (Mapara et al., 2015). To restrict the entry of antibiotics inside the cells, bacteria have evolved the mechanisms, which changes the cellular permeability. This usually includes reduced or differentially regulated expression of porins; the outer membrane channels used by the antibiotics to cross the outer membrane (Pagès et al., 2008). Several kinds of porin were identified and classified based on their structure, selectivity as well as regulation of their expression levels. Some of the well-characterized bacterial porins include OmpC, OmpF and PhoE from *E. coli* and OprD from *P. aeruginosa* (Nikaido, 2003). On the other hand, bacterial efflux pumps have emerged as vital transporters in resistant strains, which transport the antibiotic molecules out of the cell usually in non-specific manner and reduces the antibiotic concentration side the cell (Shriram et al., 2018). On the basis of the energy source, the bacterial efflux pumps are categorized in two super-families; 1) ATP-binding cassette (ABC) multidrug transporters, and 2) secondary transporters using proton motive force (PMF) as an energy source. The secondary transporters are further sub-classed into four families, namely 1) major facilitator superfamily (MFS), 2) resistance-nodulation-cell division (RND), 3) multidrug and toxic compound extrusion (MATE), and 4) small-MDR (SMR) family (Putman et al., 2000; Fernández and Hancock, 2012; Sun et al., 2014). Many of these classes are well characterized in various pathogens with respect to their substrate specificities (reviewed by Shriram et al. (2018)). Some of the most widely analyzed bacterial efflux pump system includes MexAB-OprM from *P. aeruginosa*, AcrAB-TolC from *E. coli*, NorA from *S. aureus* and KnpEF from *K. pneumoniae*. The pathogens over-representing the efflux pumps on their membranes facilitates the effective elimination of the antibiotics from the inner cellular compartment and subsequently retracts the effective interaction between antibiotics and their targets to attain the resistance. The reduced rate of the antibiotic influx via altering the cell permeability through regulated porin presentations on cell membrane and increasing the efficacy of antibiotic efflux via active efflux pump is effectively causing the development of antibiotic resistance.

### Modification of the Antibiotic Targets

Another strategy employed by the MDR pathogens to exterminate the effects of antibiotics includes the modification of the antibiotic target sites, which prevents the optimal interaction between antibiotics and targets. Such types of target modifications may include 1) enzymatic modification of the binding sites on the targets (addition of methyl group), 2) replacement of the original targets and 3) mutation (point) in the target-coding genes. Irrespective of the type of target modification, the ultimate result is decreased affinity of the antibiotics towards the targets and subsequent development of the resistance (Munita and Arias, 2016). For instance, the target modification resistance has been studied during the development of rifampin resistance due to the point mutation in *rpoB* subunit of the RNA polymerase (Floss and Yu, 2005). The development of macrolide resistance was observed due to the methylation of macrolide-target in the ribosome. The process is catalyzed by an enzyme encoded by the *erm* genes (erythromycin ribosomal methylation) (Leclercq, 2002), and methicillin resistance in *S. aureus* due to the acquisition of an exogenous penicillin-binding proteins (PBP2a) encoded by *mecA* gene (Moellering, 2012).

Additionally, the acquisition of foreign genetic material by bacteria via horizontal gene transfer facilitates the antibiotic resistance development event. The antibiotic resistance related genes have been observed to be transferred from resistant to non-resistant strains via horizontal gene transfer events such as transformation, transduction or conjugation. In such kind of resistance development, mobile genetic elements as well as integrons plays pivotal role in effective transfer of resistance related genes (Thomas and Nielsen, 2005; Manson et al., 2010).

## Horizontal Gene-Transfer (HGT) and Mobile Genetic Elements (MGEs)

The horizontal gene transfer is one of the effective processes of microbial evolution and adaptation which allows rapid procurement of new metabolic abilities and microbial phenotypes. During the process of horizontal gene transfer, there is an inter-generational transfer of the genetic material (Wiedenbeck and Cohan, 2011; Hall et al., 2017). Interestingly, the horizontal gene transfer can lead to prompt adaptation of pathogens to the environment harboring high antibiotic levels (Saak et al., 2020). These adaptations can be in the form of acquisition of active of virulence or new metabolic pathways. The genes responsible for such events are usually present of the mobile genetic elements. These mobile genetic elements are the DNA pieces with an ability to move within the host genome or between the genomes of two different (donor and recipient) cells. The bacterial mobile genetic elements are represented by plasmids, phages as well as transposable elements which can be differed based on their mode of mobilization as well as structure (Saak et al., 2020).

The canonical mechanisms of horizontal gene transfer include transformation, transduction, conjugation and transposition. During the transformation event, the DNA is taken up by the competent cells via proteinaceous DNA uptake machinery (Claverys et al., 2009; Dubnau and Blokesch, 2019). On the other hand, the transposition is achieved by the transposable elements which transpose across the different locations within the genome (Siguier et al., 2017). In bacteria, it is well established that the resistant conferring transposons can integrate themselves in mobile genetic elements such as plasmids and can get shared via horizontal gene transfer (Hedges and Jacob, 1974). Integrons are yet another type of elements that can capture resistant conferring genes. They enable the expression of enable genes via site-specific recombination downstream of a resident promoter (Mazel, 2006). During conjugation, the genetic materials such as extra-chromosomal circular plasmids, linear plasmids or conjugative transposons is transferred through a mating junction formed between two bacterial cells via a physical contact (Wozniak and Waldor, 2010; Dib et al., 2015; Delavat et al., 2017). Lastly, the transduction can occur due to the defects during DNA packaging into viral particles (general transduction), or inaccurate prophages excision from genomes (specialized transduction) (Chiang et al., 2019).

Apart from these canonical mechanisms, new mechanisms of horizontal gene transfer are emerging. For example, gene transfer agents such as the elements common for transduction and transformation were discovered (Westbye et al., 2017). The membrane vesicles formed from the host cell membrane have also displayed the ability to carry the genomic material (chromosomal/plasmid/phage) (Grüll et al., 2018). Such vesicle mediated is considered as vesiduction mode of horizontal gene transfer (Soler and Forterre, 2020). Recently, two new conjugative methods namely, mycoplasma chromosomal transfer (Dordet-Frisoni et al., 2019) and distributive conjugal transfer (Gray and Derbyshire, 2018) were described. Another emerging way is referred as transjugation which is hybrid machinery found in transformation and conjugation (Blesa et al., 2015; Blesa et al., 2017). These transferring events are even more complex as the mobile genetic elements themselves can be incorporated inside one-another. For instance, transposons can be integrated into plasmids or even into other transposons (Sheppard et al., 2016).

All these mechanisms contribute heavily during the horizontal transfer of the genes responsible for the spread of antibiotic resistance. Additionally, the emergence of new machineries involved in the horizontal gene justifying the rapidness in the spread of antibiotic resistance not only in nosocomial infections but also in the environment. The diversity in the mechanisms deciphering the spread of antibiotic resistance related genes and the rapid spread in resistance vindicates the severity of this issue. Hence, there is an urgent requirement of the new/innovative therapeutics to slow or stop the spread of antibiotic resistance.

## Phytochemicals for Combating Antibiotic Resistance

**FIGURE 2:**
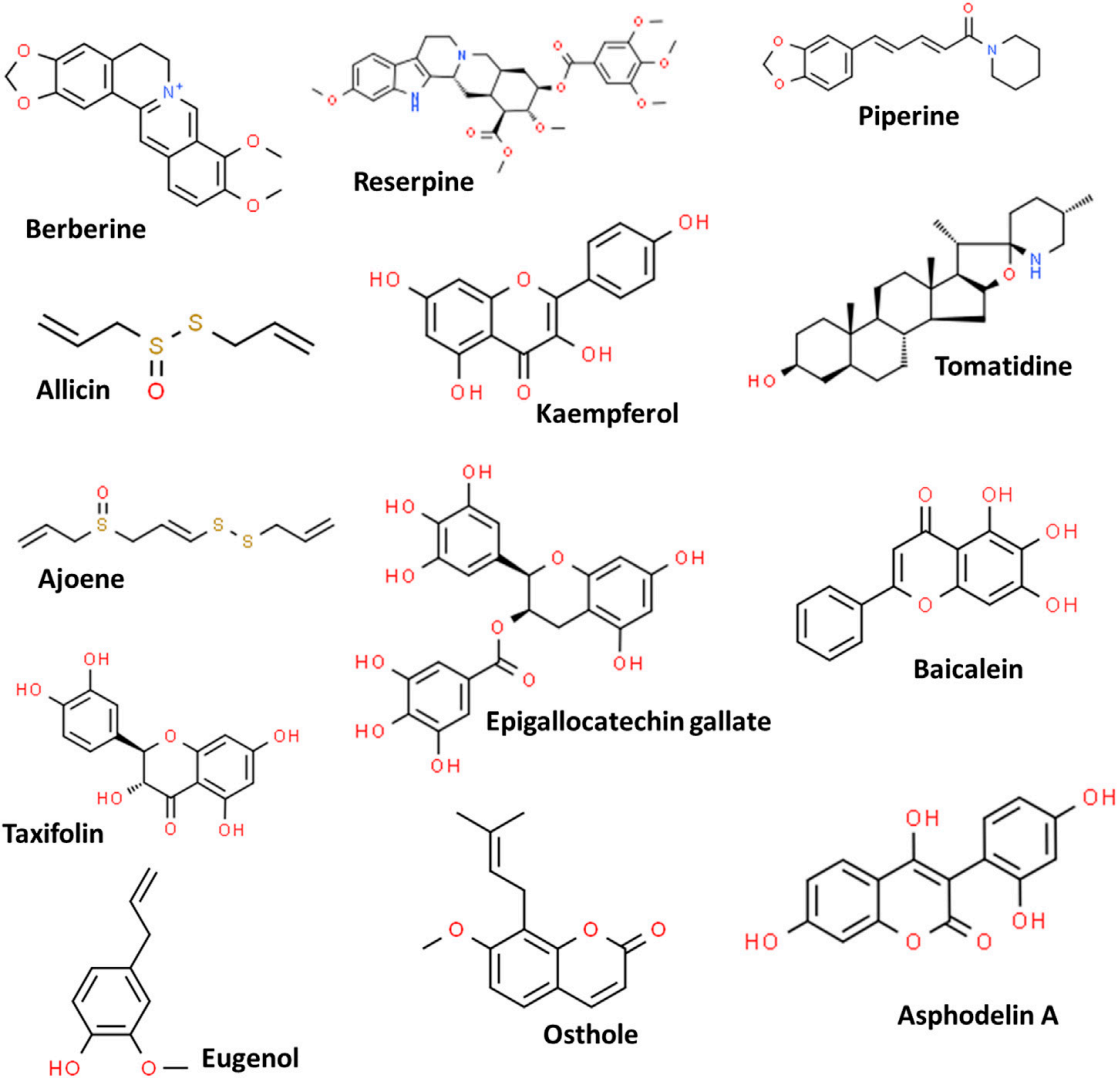
Some potent phytochemicals used against the multidrug-resistant pathogens.

Plants are known for their therapeutic potentials, initially in the form of crude extracts or powders and further as purified products (pure active phytomolecules) due to their high potencies (Samy and Gopalakrishnakone, 2010). This can be traced back to 1803 from the report on the isolation of morphine from *Papaver somniferum* (Schmitz, 1985). Due to their effective applications as food supplements, herbal medicines, cosmetics as well as antibacterial activities, the phytochemicals have gained interest in the scientific community; and numerous phytochemicals have been validated for their antimicrobial potentials including against MDR strains (Shriram et al., 2018; Anand et al., 2019; Yu et al., 2020; Mohammed et al., 2021). However, despite the reports on many phytochemicals, few have been approved by the Food and Drug Administration (FDA) such as codeine, capsaicin, reserpine, paclitaxel, colchicine, to name a few (Kongkham et al., 2020). Some important phytochemicals and their chemical structures are depicted in Figure 2.

The phytochemicals when applied in form of extracts have been demonstrated to impose inhibitory effects against clinical isolates. For example, Dahiya and Purkayastha (2012) tested various solvent-based extracts of medicinal plants including Lemongrass, Neem, Aloe vera, Oregano, Rosemary, Thyme, Tulsi and Bryophyllum against ten MDR clinical isolates (including Gram-positive and Gram-negative bacteria). The study correlated the flavonoids and tannins present in a methanolic extract with the antimicrobial activities against methicillin-resistant *S. aureus*. Four different extracts of *Lawsonia inermis* were tested successfully against Gram-negative (*E. coli, S. typhi, Klebsiella* spp*., S. sonnei*) and Gram-positive (*B. subtilis, S. aureus, S. epidermidis*) clinical isolates. Interestingly, not all these extracts induced any sign of toxidrome (Gull et al., 2013). Various solvent extracts of medicinal plants like *Curcuma longa*, *Zingiber officinale* and *Tinospora cordifolia* showed the effective killing of clinical microbes including *S. aureus, P. aeruginosa, K. pneumoniae, E. coli, B. subtilis* and *P. mirabilis* (Chakraborty et al., 2014). A summarized list of the antimicrobial potentials of medicinal plant resources is given in Table 1.

**TABLE 1:** A summarized list of phytochemicals, their chemical class, source and mode of action against pathogenic strains.

Chemical Class	Phytochemical	Source	Target pathogen	Mode of action	Reference
Alkaloid	Berberine	*Berberis* species	*Escherichia coli*	Cell division/Protein/DNA synthesis inhibitor	Boberek et al. (2010)
	Conessine	*Holarrhena antidysenterica*	*Pseudomonas aeruginosa*	Efflux pump inhibitor	Siriyong et al. (2017)
	Lysergol	*Ipomoea muricata*	*Escherichia coli*	Efflux pump inhibitor	Maurya et al. (2013)
	Reserpine	*Rauwolfia serpentina*	*Staphylococcus* sp., *Streptococcus* sp.	Efflux pump inhibitor	Sridevi et al. (2017)
	8-epidiosbulbin E-acetate	*Dioscorea bulbifera*	*Escherichia coli, Enterococcus faecalis,*	Plasmid curing	Shriram et al. (2008)
			*Pseudomonas aeruginosa, Shigella sonnei*		
	Tomatidine	*Solanaceous* plants	*Listeria*, *Bacillus* and *Staphylococcus* spp.	ATP synthase inhibitor	Boulet et al. (2018), Guay et al. (2018)
Organosulfur	*Allicin*	*Allium sativum*	*Staphylococcus epidermidis,*	Sulfhydryl-dependent enzyme inhibitor, DNA/protein synthesis inhibitor	Reiter et al. (2017)
			*Pseudomonas aeruginosa,*		
	Ajoene	*Allium sativum*	*Campylobacter jejuni,*	Sulfhydryl-dependent enzyme inhibitor	Rehman and Mairaj (2013)
			*Staphylococcus,*		
			*Escherichia coli*		
	Sulforaphane	*Diplotaxis harra*	*Escherichia coli*	Membrane destruction, ATP synthase inhibitor, DNA/protein synthesis inhibitor	Wu et al. (2012)
Phenolic compounds	Baicalein	*Scutellaria baicalensis*	Methicillin-resistant *Staphylococcus aureus*	Efflux pump inhibitor	Chan et al. (2011)
	Kaempferol	*Alpinia calcarata*	Methicillin-resistant *Staphylococcus aureus*	Efflux pump inhibitor	Randhawa et al. (2016)
	Resveratrol	*Vitis vinifera*	*Campylobacter jejuni*	Efflux pump inhibitor	Klančnik et al. (2017)
	Taxifolin	*Allium cepa*	*Enterococcus faecalis*	Beta-Ketoacyl acyl carrier protein synthase inhibitor	Jeong et al. (2009)
Coumarin	Aegelinol	*Ferulago campestris*	*Enterobacter aerogenes, Salmonella enterica serovar*	DNA gyrase inhibitor	Basile et al. (2009)
			*Typhi, Enterobacter cloacae, Staphylococcus aureus*		
	Asphodelin A	*Asphodelus microcarpus*	*Staphylococcus aureus, Escherichia coli, Pseudomonas aeruginosa*	DNA gyrase inhibitor	El-Seedi (2007)
	Galbanic acid	*Ferula szowitsiana*	*Staphylococcus aureus*	Efflux pump inhibitor	Bazzaz et al. (2010)
	Osthole	*Prangos hulusii*	*Bacillus subtilis, Staphylococcus aureus,*	DNA gyrase inhibitor	Tan et al. (2017)
			*Klebsiella pneumoniae,*		
Terpene	Carvacrol	*Thymus capitatus*	*Escherichia coli, Enterobacter aerogenes, Staphylococcus aureus, Pseudomonas*	Cell membrane disruption	Althunibat et al. (2016)
			*Aeruginosa*		
	Cinnamaldehyde	Essential oils	*E. coli and S. aureus*	Cell membrane disruption	Zhang et al. (2015)
	Eugenol	Essential oils	Methicillin-resistant *Staphylococcus aureus*	Cell membrane disruption	Yadav et al. (2015)
	Farnesol	Essential oils	*Staphylococcus aureus*	Cell membrane disruption	Togashi et al. (2010)

Apart from the extracts representing the complex mixture of many phytochemicals in a single formulation, a number of the single active principles in their purified forms have been tested for their potential against MDR pathogens along with the bacterial mechanisms targeted by them. Such pure compounds have demonstrated their activity either with their sole application or with their synergistic application with the standard antibiotics. For instance, Berberine (isoquinoline alkaloid) found in roots/stem of *Berberis* species, has exhibited antibacterial activity, which can be correlated with the action of berberine against DNA intercalation, RNA polymerase, gyrase /topoisomerase, as well as the inhibition of cellular division (Iwasa et al., 2001; Yi et al., 2007; Domadia et al., 2008). The aminothiazolyl berberine derivatives have also shown their potential antimicrobial activity against clinically resistant *A. baumannii* (Gao et al., 2018). This activity was attributed to the inhibition of DNA gyrase via hydrogen bonding. Reserpine (indole alkaloid) from the plant *Rauwolfia serpentina* has also exhibited potent efflux pump inhibitory activity (Sridevi et al., 2017). Piperine (alkaloid) from *Piper nigrum* and *Piper longum* has displayed the inhibitory action against methicillin-resistant *S. aureus* when co-administered with antibiotics such as ciprofloxacin or gentamicin. This inhibitory effect of piperine was attributed to its efflux pump inhibitory activity against the NorA efflux pumps in the MDR pathogens under consideration (Kumar et al., 2008; Khameneh et al., 2015). Another compound, tomatidine (steroidal alkaloid) from Solanaceous plants (tomato, potato, eggplant etc.), which has demonstrated the antibacterial potential against drug-resistant *S. aureus* synergistically with aminoglycosides (Mitchell et al., 2012). Tomatidine has also proved its role as antibiotic potentiator for various antibiotics, including ampicillin, cefepime, gentamicin, and ciprofloxacin against pathogens including *S. aureus, P. aeruginosa*, and *Enterococcus faecalis* (Soltani et al., 2017). Allicin (diallyl thiosulfinate, organosulfur compound) is found in the plants from the family *Alliaceae*, especially fin garlic (*Allium sativum*). The antibacterial potential of this phytochemical has been acknowledged for a long time and its antibacterial activity has been proved against pathogens such as *S. epidermidis*, methicillin-resistant *S. aureus*, *P. aeruginosa* and the periodontitis causing oral pathogens as well as human lung pathogens. Allicin also reported to potentiating the activities of antibiotics ciprofloxacin, tobramycin, and cefoperazone against *P. aeruginosa* (Cai et al., 2008; Reiter et al., 2017). The possible mechanism of action of allicin is postulated as inhibition of sulfhydryl-dependent enzymes allicin, such as RNA polymerase, thioredoxin reductase and alcohol dehydrogenase (Lanzotti et al., 2014). The isothiocyanates (volatile organosulfur compounds) isolated from the *Armoracia rusticana* have displayed the strong activity against oral pathogens as well as bactericidal activity against *H. pylori* via inhibiting the urease activity and decreasing the inflammatory factor of Helicobacter infection (Fahey et al., 2013; Park et al., 2013).

The phenolic compound resveratrol is known for its efflux pump inhibitory activities and is capable of blocking the *CmeABC* type efflux pumps in *Campylobacter jejuni* (Klančnik et al., 2017). Kaempferol (flavonoid) and its natural glycoside derivative from *Persea lingue* proved to increase the antimicrobial potential of ciprofloxacin in a NorA overexpressed *S. aureus* strain (Holler et al., 2012). Chalcones and catechin gallates are yet another group of phytochemicals with potential efflux pump inhibitory activities (Belofsky et al., 2004; Gibbons et al., 2004). The epigallocatechin gallate from green tea inhibits the DNA gyrase (B subunit) at the ATP binding site (Gradišar et al., 2007).

**FIGURE 3:**
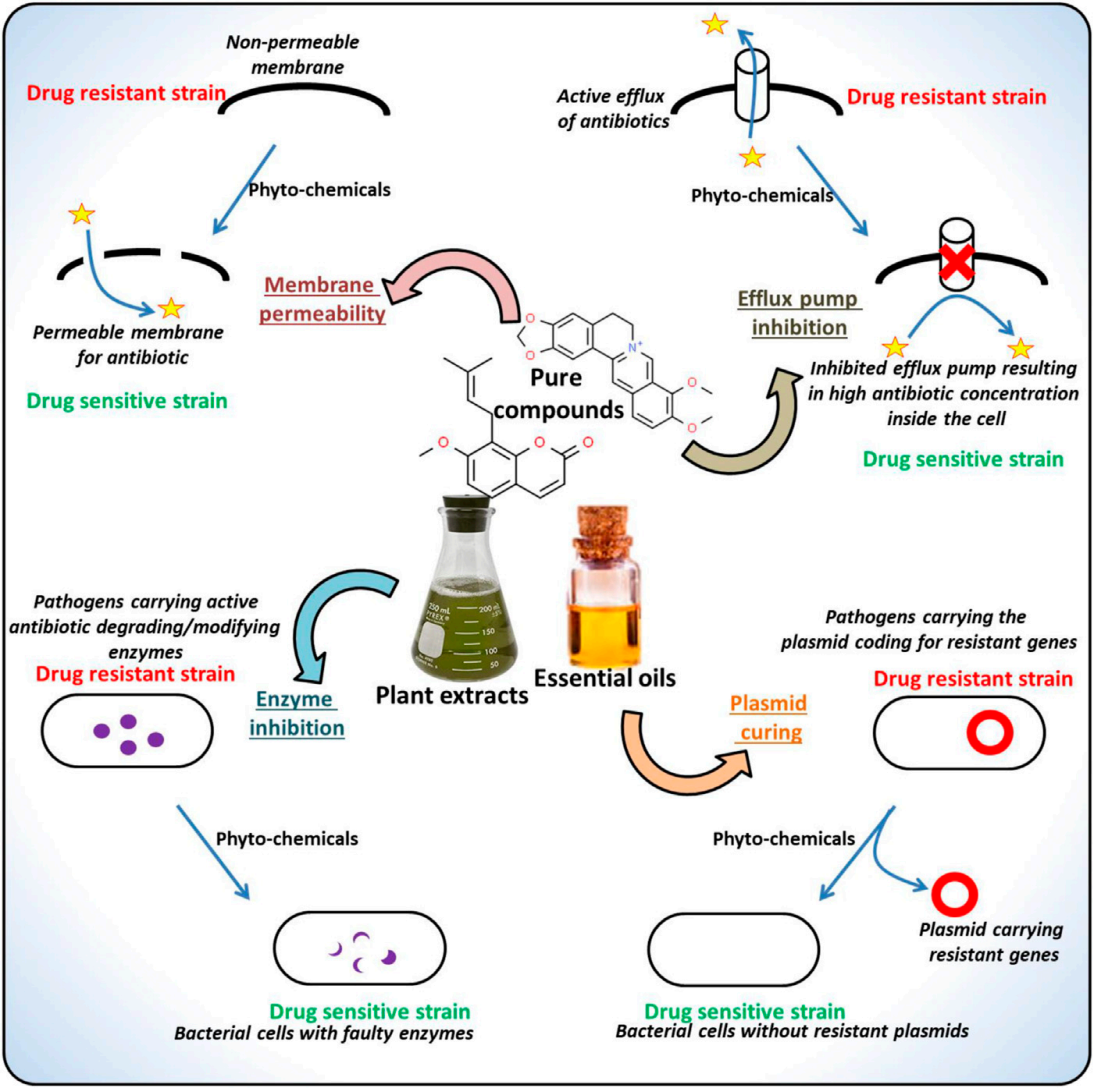
Major mechanisms underlying microbial drug-resistance targeted by the phytochemicals.

The frequent location of the genes conferring the antibiotic resistance is a plasmid. These plasmids are usually characterized as self-replicating, circular DNAs coding for various gene groups with different functionality. The phytochemicals targeting such plasmids have also been reported (Buckner et al., 2018). For instance, the 8-epidiosbulbin-E-acetate from *Dioscorea bulbifera* proved to cure the antibiotic-resistant R-plasmids of clinical isolates of *E. faecalis*, *E. coli*, *Shigella sonnei* and *P. aeruginosa* with 12–48% curing efficiency (Shriram et al., 2008). There are several more instances of the successful application of the phytochemicals against various MDR pathogens, examples of the phytochemicals along with their potential inhibitory activities are listed in Table 1 and the drug-resistant determinants targeted by the phytochemicals are depicted in Figure 3. The following sections discuss the major types of phytochemicals including alkaloids, phenolics, flavonoids and essential oils, their antimicrobial potentials against drug-resistant microbes and the mode of actions.

### Alkaloids

Alkaloids are organic nitrogen heterocyclic compounds with a wide range of chemical structures. Nicotine, morphine, caffeine, and mescaline are examples of alkaloids, which are base-forming water-soluble salts derived from an amino acid (Compean and Ynalvez, 2014). They are found in over 300 plant families, with unique compounds found in just a few of them (e.g., hyoscyamine in Solanaceae) (Evans, 2009). Alkaloids can be found in several parts of the plant, but certain compounds are restricted to a particular type (for example, quinine in cinchona tree bark) (Robbers et al., 1996). Alkaloid-rich biorational plant extracts from various families, including Amaryllidaceae, Burseraceae, Capparaceae, Mimosaceae, Vitaceae, and Tiliaceae, have remarkably demonstrated antibacterial activity against mycobacteria (*M. fortuitum*, *M. tuberculosis*, and *M. smegmatis*), *E. coli*, *S. aureus*, *S. Typhimurium*, *K. pneumonia* and *P. aeruginosa* (Thawabteh et al., 2019; Omar et al., 2021).

Alkaloids have shown broad-spectrum antimicrobial activities, and several studies have suggested that these compounds could represent an important role in tackling pathogenesis of a variety of infection agents (Cushnie et al., 2014; Casciaro et al., 2020). Alkaloids are known to target major drug-resistance determinants, especially the powerful EPs. A piperidine-type alkaloid (Piperine) isolated from *Piper nigrum* and *P. longum*, prevented the growth of a mutant *S. aureus* and significantly decreased the MIC values for *S. aureus* when co-administered with ciprofloxacin (Khan et al., 2006). Co-loaded piperine and gentamicin nanoliposomes were tested for antibacterial activity and EP-inhibitory action in MRSA and the results demonstrated effectiveness in reducing MRSA infection (Khameneh et al., 2015). The use of piperine as an EP inhibitor (EPI) was investigated, and the findings revealed that this compound affected MRSA and *S. aureus* NorA (388 amino acid protein) EPs (Kumar et al., 2008; Khameneh et al., 2015). Besides, the synthetic piperine analogs including 5-(2,2-dimethyl-chroman-6-yl)-4-methyl-penta-2,4-dienoic acid ethyl ester 5 and 5-(2,2-Dimethyl-chroman-6-yl)-4-methyl-2E,4E-pentadienoic acid pyrrolidide have also been proved their potential as inhibitors of the NorA overexpressing *S. aureus* (Chopra et al., 2019).

Berberine, an isoquinoline alkaloid from Berberis species roots and stem bark, besides being the main active ingredient in *Cortex phellodendri* and *Rhizoma coptidis*, has been used in herbal medicine for centuries. This phytochemical has antibacterial, antifungal, antiprotozoal, and antiviral properties. The antibacterial action mechanism of Berberine includes DNA intercalation, targeting RNA polymerase, gyrase, and topoisomerase IV, besides cell division inhibition (Yi et al., 2007; Kang et al., 2015; Peng et al., 2015). In addition, its antibacterial properties are also associated with the inhibition of the FtsZ (Filamenting temperature-sensitive mutant Z) protein, cell division protein (Boberek et al., 2010). FtsZ inhibitors could be considered a novel class of antibacterial agents with the ability to demonstrate broad-spectrum activity. This compound has also been shown to inhibit bacterial cell function through a variety of mechanisms, including cell structure damage, protein and DNA synthesis inhibitors, which ultimately leading to bacterial death (Feng et al., 2016). In addition, synthetic derivatives, such as 9, 13-disubstituted berberines, have also displayed potent antistaphylococcal activities (Wang F. et al., 2017). Ungeremine, an isoquinoline alkaloid that was discovered in a methanol extract of *Pancratium illyricum* L. bulbs, and has been shown to possess antibacterial properties. By targeting and inhibiting the bacterial topoisomerase IA, this compound can cause a significant increase in DNA cleavage (Casu et al., 2011; Schrader et al., 2013).

Quinoline alkaloids (such as masculine, kokusagine and dictamnine) isolated from the stem bark of *Teclea afzelii* has shown strong antibacterial activities (Kuete et al., 2008). Quinolone alkaloids, natural and synthetic, have been reported to inhibit type II topoisomerase enzymes, and as a result, inhibited DNA replication (Heeb et al., 2011). Reserpine, an indole alkaloid obtained from the *Rauwolfia serpentina* plant and is a well-known natural compound with strong EPI activity (Abdelfatah and Efferth, 2015). It has been demonstrated that the overexpression of EP was the key mechanism of resistance to fluoroquinolones in resistant *Stenotrophomonas maltophilia* and the addition of reserpine reduced the antibiotic resistance capabilities (Jia et al., 2015). Tomatidine, a steroidal alkaloid found in solanaceous plants such as eggplant (*Solanum melongena*), potato (*Solanum tuberosum*), and tomato (*Solanum lycopersicum*), and has been shown to have significant antibacterial activity against *S. aureus* whether used alone or in conjunction with aminoglycosides (Jiang et al., 2016).

Further, several studies have demonstrated the synergistic effects of alkaloids when used with antibiotics, for instance, tomatidine and aminoglycosides against drug-resistant *S. aureus* strains (Mitchell et al., 2012). Tomatidine could be accounted as an antibiotic potentiator for many antibiotics like ampicillin, cefepime, gentamicin, and ciprofloxacin, and against both Gram-negative and Gram-positive bacteria like *P. aeruginosa*, *S. aureus*, and *Enterococcus faecalis* infections (Soltani et al., 2017). Chanoclavine, a tricyclic ergot alkaloid isolated from the seeds of *Ipomoea muricata* shown synergistic effects when combined with tetracycline in the treatment of MDR *E. coli*. EP, which appears to be similar to the ATPase-dependent ones, has been discovered to be inhibited by this compound (Dwivedi et al., 2019). *Holarrhena antidysenterica* is a member of the Apocynaceae family that has historically been used to treat a variety of ailments, including fever, diarrhea, bacterial infections, and dysentery (Siriyong et al., 2017). The barks of *H. antidysenterica* are full of alkaloids especially the steroidal alkaloid conessine which is responsible for its therapeutic potential (Zahara et al., 2020). A previous *in vitro* study demonstrated that conessine restores the inhibitory activity of rifampicin and novobiocin against drug-resistant *Acinetobacter baumannii* (Phatthalung et al., 2012; Chusri et al., 2014; Siriyong et al., 2016). In comparison to the constituent monotherapies, combination therapies of steroidal alkaloids or conessine with levofloxacin improved bacterial inhibition *in vitro* and restored antibiotic efficacy *in vivo* (Siriyong et al., 2018). Furthermore, this compound showed EPI activity against AdeIJK EP, which is involved in the efflux of several antibiotics in *A. baumannii* (Damier-Piolle et al., 2008; Siriyong et al., 2016). Sanguinarine is a benzophenanthridine alkaloid obtained from the rhizomes of *Sanguinaria canadensis*, has long been known to have broad antimicrobial and anti-inflammatory properties. The antibacterial activity of this alkaloid is mediated by the disruption of bacterial FtsZ Z-ring formation and inhibition of bacterial cytokinesis (Kelley et al., 2012). A combination of sanguinarine, vancomycin, and EDTA as well as a combination of sanguinarine, streptomycin and EDTA were tested against both Gram-negative and Gram-positive bacteria, including MDR phenotypes, in two recent studies (Hamoud et al., 2014, 2015). These experiments revealed that sanguinarine has potent antibacterial activity against all strains tested; additive and synergistic effects were observed for all sanguinarine + sanguinarine and EDTA + vancomycin + EDTA combinations against gram-negative bacteria. Except for MRSA, the combination of sanguinarine + EDTA + streptomycin demonstrated synergistic activity against almost all strains. Further, the activity of sanguinarine against MRSA strains was investigated besides its mechanism of action. Treatment of bacteria with sanguinarine has been shown to cause the release of membrane-bound cell wall autolytic enzymes, resulting in cell lysis; however, transmission electron microscopy (TEM) of MRSA has revealed alterations in the formation of septa. Overall, the results revealed a possible sanguinarine mechanism of action against MRSA by compromising the cytoplasmic membrane (Obiang-Obounou et al., 2011). Moreover, this compound was proposed to display antimycobacterial properties against *Mycobacterium aurum* and *Mycobacterium smegmatis*, two model mycobacteria species (Newton et al., 2002).

### Phenolic Compounds

Plant phenolic compounds are regarded as crucial natural bioactive molecules due to their wide-spectrum, striking pharmacological activities. Plant phenolics have an aromatic ring with one or more hydroxyl groups in its molecular structure, and it can be a simple or polymerized molecule. Plant phenolics are classified into many groups based on their structural characteristics, with flavonoids, phenolic acids, and non-flavonoids being the most common (de Souza et al., 2019). They have proven potent in the treatment of many chronic diseases, including bacterial infection, cancer, diabetes, and cardiovascular disease, in terms of human health (Del Rio et al., 2013; Zacchino et al., 2017; Lima et al., 2019). Plant-phenolics have been shown to exhibit antimicrobial properties against a wide range of microorganisms, sensitize MDR strains to bacteriostatic or bactericidal antibiotics, and are promising natural antimicrobial weapons (Miklasińska-Majdanik et al., 2018). Due to their direct antimicrobial action and antibiotic modulation activities, dietary polyphenols have recently been demonstrated as chemopreventive and therapeutic agents (Makarewicz et al., 2021). Phenols, including pyrogallol and catechol, demonstrated antimicrobial activity against several microorganisms, including both Gram-positive and Gram-negative bacteria. Pyrogallol, for example, had a MIC range of 2.4–2500 μg/ml and pyrocatechol had a MIC range of 4.9–312.5 μg/ml against many periodontitis-causing microorganisms (Shahzad et al., 2015). Moreover, the halogenated catechols have also demonstrated the intrinsic inhibitory potential against five MDR strains via inhibiting the bacterial fatty acid synthesis (Liu et al., 2021). Taguri et al. (2006) evaluated 22 polyphenolic compounds for antibacterial activity against Gram-negative and Gram-positive bacteria and the results showed that pyrogallol-based compounds were more effective than resorcinol or catechol (Taguri et al., 2006).

Certain phenolic acids, such as Caffeic acid, exhibit antibacterial properties. In comparison to ampicillin, which demonstrated MICs of 0.1 and 3.2 μg/ml, Caffeic acid demonstrated an MIC of 1600 μg/ml against *E. coli* and S*. aureus* (Lim et al., 2016). Moreover, gallic acid has been shown to exhibit antibacterial properties against *Enterococcus faecalis* (Aires et al., 2013). It has also showed strong antibacterial potentials against *Streptococcus pneumonia*, *P. aeruginosa*, *Moraxella catarrhalis*, *S. aureus*, *Enterococcus faecalis*, *E. coli*, and *Streptococcus agalactiae* strains (Cueva et al., 2012). Gallic acid was found to have strong antibacterial activity against *Campylobacter* and bactericidal activity against two *Campylobacter coli* strains with MIC of 61.5–125 μg/ml (Sarjit et al., 2015).

Ferulic acid and gallic acid exhibited antibacterial properties against many bacterial isolates, where Ferulic acid showed MICs of 100 μg/ml against *P. aeruginosa* and *E. coli*, and 1250 μg/ml and 1100 μg/ml against *L. monocytogenes* and *S. aureus* while gallic acid demonstrated MIC equal to 2000 μg/ml against *L. monocytogenes*, 500 μg/ml against *P. aeruginosa*, 1500 μg/ml against *E. coli*, and 1750 μg/ml against *S. aureus,* respectively (Borges et al., 2013). Both ferulic acid and gallic acid damage the cell walls of *E. coli*, *P. aeruginosa*, S*. aureus*, causing local damage and cellular material leakage (Borges et al., 2013). On the other hand, the ferulic acid derivatives, namely 4-((E)-2-(diethylcarbamoyl)vinyl)-2-methoxyphenyl acetate and (E)-methyl 3-(4-((p-tolylcarbamoyl)methoxy)-3-methoxyphenyl)acrylate have also displayed their putative activity as EPI to inhibit the growth of NorA Eps in MRSA (Sundaramoorthy et al., 2018). Caffeic acid, protocatechuic acid, vanillic acid (4-hydroxy-3-methoxybenzoic acid), *p*-hydroxybenzoic acid or 4-Hydroxybenzoic acid, *p*-coumaric acid, syringic acid, and ferulic acid, which are found in wild polish mushrooms, displayed intermediate antibacterial activity against a variety of Gram-negative and Gram-positive bacteria, with MIC ranging from 156 to 5000 μg/ml (Nowacka et al., 2015). *Galla rhois* synthesizes major metabolites called methyl gallate, which demonstrated anti-*Salmonella* activity against multiple *Salmonella* strains with MIC ranging from 3.9 to 125 μg/ml (Choi et al., 2014). With MIC of 30 μg/ml, methyl gallate showed strong antibacterial activity against *Shigella flexneri* and *E. coli* (Madikizela et al., 2013). Ellagic acid, caffeic acid, hyperoside, ferulic acid, (+)-catechin, and sophorin (rutin) were found to be abundant in three *Potentilla* species, which were screened for antibacterial activities. With MIC values of 0.78–6.25 mg/ml, *P. fruticosa* demonstrated strong inhibitory effects against the fungus *Candida albicans* along with Gram-negative and Gram-positive bacteria (Wang et al., 2013).

The combination of gallic acid (10 μg/ml) and isoquercitrin (10 μg/ml) was effective in inhibiting *S. aureus* growth, despite their individual MIC being 10 times higher (Soberón et al., 2014). The ethyl acetate (EtOAc) fraction from the ethanol extract of *Searsia chirindensis* (*Rhus chirindensis*) which included myricetin-3-O-arabinopyranoside, methyl gallate, quercetin-3-O-arabinofuranoside, myricetin-3-O-rhamnoside, and kaempferol-3-O-rhamnoside showed the most active antibacterial fraction. The MICs of these compounds against *E. coli*, *S. aureus*, *Campylobacter jejuni*, and *S. flexneri* ranged from 30 to 250 μg/ml (Madikizela et al., 2013).

### Flavonoids

Plant flavonoids are the 2-phenyl-benzo-γ-pyrane nucleus with two benzene ring-containing plant phenolic compounds with promising antimicrobial/antimicrobial-potentiator activities. Many classes of flavonoids, such as flavonols, flavanols, flavanones, isoflavonoids, chalcones and dihydrochalcones have been identified as allelochemicals, which inhibits the microbial growth (reviewed by Górniak et al., 2019). Along with these chemicals, the chalcone derivatives such as fluorinated-/chlorinated-/steroidal-/ferrocene-chalcones have also displayed potent antimicrobial activities against various MDR strains (Xu et al., 2019). Cushnie et al. (2008) demonstrated the catechin mediated membrane disruption in methicillin-resistant *Staphylococcus aureus* (MRSA) which causes potassium leakage and successive loss of the cell membrane integrity. Similarly, the investigation by Budzyńska et al. (2011) suggested the lipophilic nature of the 3-arylideneflavanones, which causes the aggregation of the bacterial cells and alters the membrane integrity and making the membranes more permeable in *S. aureus* and *E. faecalis* clinical isolates. Flavonoids are also proved as inhibitors of the quorum sensing and biofilm formation. The quercetin-mediated inhibition of biofilm formation was observed in *P. aeruginosa* PAO1 with reduced expression levels of the quorum sensing related genes (*lasI*, *lasR*, *rhlI* and *rhlR*) along with reduced production of virulence factors such as elastase, protease, and pyocyanin (Ouyang et al., 2016). Additionally, the antimicrobial activities of flavonoids have also displayed the association with nucleic acid synthesis. For examples, the flavonoids such as chrysin and kaempferol restrict the DNA gyrase (essential enzyme in DNA replication) activity in *E. coli* (Wu et al., 2013). Similarly, another important element during the DNA replication, the helicases (DnaB and RecBCD) are also proved to be inhibited by the flavonoids such as morin and myricetin (Xu et al., 2001). Apart from the direct antibacterial activities of flavonoids, there are also promising results indicating the role of flavonoids as resistant-reversal agents. For instance, Christena et al. (2015) demonstrated the synergistic role of pinostrobin-a with antibiotic ciprofloxacin during the growth inhibition in resistant strains of *P. aeruginosa* and *E. coli*, where pinostrobin displayed the efflux pump inhibition activity against the NorA efflux pumps. Likewise, the aglycone flavonoids such as myricetin, hesperetin and phloretin displayed the biofilm formation inhibition, which was postulated to the disturbed functioning of the msrA and norA efflux systems in *Staphylococcus* strains (Lopes et al., 2017). Lin et al. (2005) have shown the activity of twelve different flavonoids against the ESBL producing resistant strains of *K. pneumoniae*. Collectively this specifies the antimicrobial and resistant reversal potential of the flavonoids.

### Essential Oils

Essential oils (EOs) represent a mixture of several low mass plant natural products or phytochemicals. EOs are well-documented for their strong antimicrobial potentials and are used often in traditional medicinal practices (Yu et al., 2020). These are lipophilic, volatile plant products that can be extracted from various plant parts, mainly flowers and fruits. However, their spread is limited to only a number of plant families. They are derived mainly from terpenes and terpenoids, and aromatic, aliphatic aldehydes and phenols. Besides, short-chain aliphatic hydrocarbon derivatives also constitute EOs (Turek and Stintzing, 2013). EOs are looked upon as a pool of antimicrobial agents (Solórzano-Santos and Miranda-Novales, 2012; Lahmar et al., 2017).

EOs have been well-documented for their striking antibacterial activities against both Gram-positive and negative pathogens (Yap et al., 2014). Interestingly, EOs have shown both direct-killing (bactericidal) and re-potentiating or re-sensitizing of antibiotics potentials against pathogenic microbes (Brehm-Stecher and Johnson, 2003; Mayaud et al., 2008). However, considering the vulnerability of EOs for conversion or degradation events, their stability is decisive for their quality and pharmacological potencies (Turek and Stintzing, 2013). But EOs are often reported to be safe for consumption as well as for vital host tissues (Helander et al., 1998).

In last few years, several authors have reported antimicrobial activities of EOs from different plants against several antibiotic-resistant strains. Naveed et al. (2013) reported the EOs of Cinnamomum verum *barks exhibit* strong antibacterial activities against MDR phenotypes of *S. typhi*, *E. coli* and *S. aureus*. Similarly, Sharopov et al. (2015) observed strong activities of *Origanum tyttanthum* EOs against MRSA. Similarly, *Mentha piperita, Coriandrum sativum*, and *Pimpinella anisum* EOs also exhibited antimicrobial potentials against *S. aureus* and *E. coli* (Bazargani and Rohloff, 2016). The EOs of *Petroselinum crispum* and *Ocimum basilicum* were found to be potent antimicrobials against *Vibrio* strains (Snoussi et al., 2016). *Pogostemon heyneanus* and *Cinnamomum tamala* EOs were found to have anti-biofilm and anti-virulent properties against MRSA strains (Rubini et al., 2018). Carum carvi, *C*. sativum, Cuminum cyminum EOs were reported to have notable antibacterial activities against *E. coli* and *S. aureus* (Khalil et al., 2018). Bučková et al. (2018) reported striking antimicrobial activities of EOs from several plants such as oregano, thyme, and tea tree beside others against drug-resistant strains of *P. aeruginosa*, *E. coli*, *Enterobacter cloacae*, *Morganella morganii* and *Proteus mirabilis* with considerably low MIC (0.005–0.5%).

In addition to direct bacterial killing (bactericidal) potentials, plant EOs have also been explored recently for their actions as adjuvants with antibiotic for drug-resistance-reversal or re-sensitizing/re-potentiating potentials. For instance, Benameur et al. (2019) assessed the susceptibility of *bla*ESBL producing Enterobacteriaceae to Slovakian *Thymus vulgaris* EOs with/out antibiotic (cefotaxime), and observed a synergistic interaction between EOs and the antibiotic against blaSHV-12 producing MDR *E. coli*. Further, the peppermint oil exhibited synergistic impacts when used with gentamicin against ESBL- and NDM-1-producing *Klebsiella pneumoniae* strains (Kwiatkowski et al., 2018).

EOs have demonstrated their potentials in targeting and/or disturbing the most prevalent drug-resistance determining mechanisms of microbes including cell wall, cell membrane and permeability, drug efflux pumps, mobile genetic elements, quorum sensing and biofilm (Yu et al., 2020). Bacterial cell membranes work as the first barrier against antimicrobial agents, which any antibiotic should overcome for its interaction with the target (Martinez de Tejada et al., 2012). Bacterial cell membrane permeability play key regulatory roles in the entry and intracellular concentrations of antibiotics (Martinez de Tejada et al., 2012; Bueno, 2016). Bacterial strains have evolved effective ways to reform their membrane permeability mainly through altering the fatty acid- and membrane proteins, in order to have a check on the cellular influx of the applied antibiotics (Burt and Reinders, 2003; Mrozik et al., 2004). However, interestingly, EOs, being hydrophobic in nature, interact with membrane lipids and mitochondria and disturb the cellular structure ultimately resulting in higher membrane permeability. Besides, owing to their lipophilic nature, EOs can impact the amount and structure of unsaturated fatty acids in bacterial cell membranes (Nazzaro et al., 2013). Because of this, bacterial cells get unable to have a check on the seepage of key cellular molecules/ions from the bacterial cells when treated with EOs (Yu et al., 2020). Several recent reports have confirmed the strong potentials of plant EOs in targeting these critical drug-resistance determinants. M. alternifolia *EOs* increased the bacterial membrane permeability (Cox et al., 2000). Wang J. et al. (2017) reported Dodartia orientalis EOs effective against *S. aureus*, *E. coli*, and Salmonella enteritidis, *as the EOs successfully* disrupted the bacterial cell structure and resisted the biofilm formation. Further, Cinnamon EOs altered the cell microstructure, membrane permeability and integrity, attributed for striking bactericidal potencies against *E. coli* and Staphylococcus strains (Zhang et al., 2015). *Ocimum gratissimum* EOs acted as permeabilizer and showed significant antimicrobial potencies against *P. aeruginosa* and *S. aureus* (Hyldgaard et al., 2012). *Coriandrum sativum* EOs disrupted the cell membrane permeability and inhibited the MDR uropathogenic *E. coli* (Scazzocchio et al., 2019). This altered membrane permeability was attributed to apparent damages to the cellular integrity and functions (Scazzocchio et al., 2019).

Similarly, EOs have been characterized by several research groups as potent bacterial drug efflux inhibitors (EPIs) (reviewed by Yu et al. (2020)). Limaverde et al. (2017) reported *Chenopodium ambrosioides* EOs as EPIs of *S. aureus*. EOs from *Salvia fruticosa*, *S. officinalis* and *S. sclarea* significantly reduced the expression levels of tet(K) gene and the reduced drug (tetracycline) efflux from tetracycline resistant *Staphylococcus epidermidis* (Chovanová et al., 2015). EOs from *C. ambrosioides* and *Thymus vulgaris* also showed notable inhibitory activities against the NorA EPs in *S. aureus* (de Morais Oliveira-Tintino et al., 2018) and ciprofloxacin resistant *S. aureus* (Salehzadeh et al., 2018). At sub-MIC levels, the norA pump showed lower EtBr efflux, along with down-regulation of *norA* gene (Salehzadeh et al., 2018). Saviuc et al. (2016) showed EPI and membrane permeabilization-mediated synergistic effects of *Rosmarinus officinalis* EOs and eucalyptol against drug-resistant *A. baumannii* and *P. aeruginosa* phenotypes. *T. broussonetii, T. maroccanus, T. pallidus* and *R. officinalis* EOs exhibited strong EPI-mediated chemosensitizers-restoring activities against MDR phenotypes of *E. coli*, *Enterobacter aerogenes*, *K. pneumoniae*, *S. enterica* serotype Typhimurium and *P. aeruginosa* (Fadli et al., 2011). Eugenol is an important antimicrobial terpene which can be found in the EOs of plants such as cinnamon or cloves. The study by Muniz et al. (2021) reported the antibacterial activity of eugenol and its derivatives including isoeugenol, allylbenzene, 4-allylanisole, and 4-allyl-2,6-dimethoxyphenol against the *S. aureus* NorA EPs.

Mobile genetic elements, in particular plasmids, are well known for their role in horizontal gene transfer of resistance genes and thus the spread of antibiotic resistance. EOs have been assessed for their bactericidal as well as resistance reversal potentials attributed mainly to their abilities to eliminate R-plasmids. Schelz et al. (2006) reported potent antibacterial and plasmid-curing activities of EOs of several plants besides purified turpentine oil against *S. epidermidis* and *E. coli* strains. Si et al. (2008) reported strong antibacterial activities of oregano EOs against MDR ESBL *E. coli* strain, and the EOs exhibited additive effects when combined with antibiotics like amoxicillin, polymycin, and lincomycin, and the activities were attributed to the plasmid affecting abilities of EOs. A combination of antibiotics and EOs of cinnamon bark, tea tree, peppermint and lavender significantly decreased the drug-resistance level in *E. coli* (Yap et al., 2013)*.* In an interesting study, Thymus vulgaris *EOs lowered* the virulence and abilities to spread the drug-resistance in *E. coli* (Skalicka-Woźniak et al., 2018).

Biofilm formation and quorum sensing are reported to be highly effective approaches evolved by the bacteria for conferring drug-resistance, its persistence and spread. Therefore, targeting bacterial biofilms and quorum sensing are emerging as effective approaches for combating drug resistance. However, eradicating or inhibiting biofilm is challenging. However, there are recent reports on anti-biofilm and anti-quorum sensing activities of EOs. For instance, Merghni et al. (2018) reported biofilm and quorum-sensing inhibitory activities of EO from *Eucalyptus globulus* against MRSA strains. Strong biofilm inhibitory activities were reported from some important individual constituents of EOs against community-associated MRSA strains (Espina et al., 2015). The *Plectranthus amboinicus* EOs also displayed striking biofilm inhibitory activities against antibiotic resistant *S. aureus* (Vasconcelos et al., 2017). Similarly, strong biofilm and quorum sensing inhibitory activities were reported from EOs of *T. daenensis* and *Satureja hortensis* against *S. aureus* (Sharifi et al., 2018)*.* A study by Alibi et al. (2020) have demonstrated the successful application of the EOs from *Cinnamomum verum*, *Thymus vulgaris* and *Eugenia caryophyllata* against to inhibit growth of 105 MDR clinical isolates via significant anti-biofilm and anti-quorum sensing activities.

## Conclusion and Future Perspectives

Antibiotic-resistant pathogens are causing major concern to the current global healthcare system. Most of these pathogens are notably developed via the nosocomial sources and the speed of progression and spread of antibiotic-resistant strains is unprecedented and extraordinarily pacey. The development of new/alternative and effective course of treatments against drug resistant pathogens is therefore emerging as a top priority globally. The active principles of plant origin, referred to as phytochemicals have emerged as an alternative to the conventional antibiotics to treat such antibiotic resistant pathogen originated infections. Many phytochemicals have demonstrated their potential as bactericidal agents or antimicrobial agents potentiating the activity of existing antibiotics (antibiotic reversal agents). These phytochemicals are proving to the promising alternatives in response to the shrinking pool of conventional antibiotics. These phytochemicals have proved to inhibit the major resistance-gaining determinant such as efflux pumps, replication machineries, cell permeability and other events vital for sustenance and resistance of the pathogen. The combinatorial application of these phytochemicals has proved well effective against antibiotic-resistant strains. These phytochemicals have displayed themselves as future drugs and knowledge that is more scientific in this regard is essential. Even though there are numerous successful examples of phytochemicals combating antibiotic resistant infections, the translational success or commercial application of such phytochemicals is very low and needs a drastic improvement. Hence, there is the utmost need for fast-track research, clinical approval and application of these phytochemicals to battle against the MDR associated clinical complications.
